# Human Islet Amyloid Polypeptide Transgenic Mice: In Vivo and Ex Vivo Models for the Role of hIAPP in Type 2 Diabetes Mellitus

**DOI:** 10.1155/2008/697035

**Published:** 2008-05-12

**Authors:** J. W. M. Höppener, H. M. Jacobs, N. Wierup, G. Sotthewes, M. Sprong, P. de Vos, R. Berger, F. Sundler, B. Ahrén

**Affiliations:** ^1^Division of Biomedical Genetics, Department of Metabolic and Endocrine Diseases, University Medical Center Utrecht, P.O. Box 85090, 3508 AB Utrecht, The Netherlands; ^2^Netherlands Metabolomics Centre, location Utrecht, P.O. Box 85090, 3508 AB Utrecht, The Netherlands; ^3^Division Laboratories and Pharmacy, Department of Endocrinology, University Medical Center Utrecht, P.O. Box 85090, 3508 AB Utrecht, The Netherlands; ^4^Department of Experimental Medical Sciences, Lund University, BMC, C 12, S-221 84 Lund, Sweden; ^5^Division of Medical Biology, Department of Pathology and Laboratory Medicine, University of Groningen, Hanzeplein 1, 9700 RB, Groningen, The Netherlands; ^6^Department of Clinical Sciences, Lund University, BMC, C 12, S-221 84 Lund, Sweden

## Abstract

Human islet amyloid polypeptide (hIAPP), a pancreatic islet protein of 37 amino acids, is the main component of islet amyloid, seen at autopsy in patients with type 2 diabetes mellitus (DM2). To investigate the roles of hIAPP and islet amyloid in DM2, we generated transgenic mice expressing hIAPP in their islet beta cells. In this study, we found that after a long-term, high-fat diet challenge islet amyloid was observed in only 4 of 19 hIAPP transgenic mice. hIAPP transgenic females exhibited severe glucose intolerance, which was associated with a downregulation of GLUT-2 mRNA expression. In isolated islets from hIAPP males cultured for 3 weeks on high-glucose medium, the percentage of amyloid containing islets increased from 5.5% to 70%. This ex vivo system will allow a more rapid, convenient, and specific study of factors influencing islet amyloidosis as well as of therapeutic strategies to interfere with this pathological process.

## 1. INTRODUCTION

Islet amyloid polypeptide (IAPP), also referred to as amylin, is a 37 amino acid protein produced in the pancreatic islet beta cells. Human IAPP (hIAPP) is implicated in the pathophysiology of type 2 diabetes mellitus (DM2) since it forms proteinaceous tissue deposits in the pancreatic islets (“islet amyloid”) [1–3]. Islet amyloid has been demonstrated in more than 80% of patients with DM2 [4, 5]. Islet amyloid formation is implicated in development of beta cell failure which, in addition to insulin resistance, is a characteristic of DM2 [6]. Overproduction of IAPP in insulin resistance may occur due to common transcription regulatory elements in the promoter regions of the IAPP and insulin genes [7], and this might underly the enhanced amyloid formation in DM2 [3]. This in turn may induce impairment of beta cell function since aggregation of hIAPP has been demonstrated to be cytotoxic [8–10]. However, involvement of islet amyloid in development of DM2 is still not firmly established. Recent data, both for hIAPP and for other amyloidogenic proteins (notably the Alzheimer's disease-related Abeta peptide), indicate that the degree of amyloid formation does not correspond with the severity of disease [11, 12]. In addition, prefibrillar aggregates of amyloidogenic proteins seem to be more cytotoxic than mature amyloid fibrils [13, 14]. To explore the potential diabetogenic effects of hIAPP and islet amyloid, we have generated transgenic mice overproducing biologically active hIAPP in the islet beta cells [15–17] (mouse IAPP does not form islet amyloid). We previously showed that hIAPP overexpression in itself does not induce hyperglycemia, hyperinsulinemia, or obesity in these mice [15]. However, when the hIAPP transgenic mice were crossbred with leptin-deficient and insulin-resistant ob/ob mice, extensive islet amyloid formation with worsening of the diabetes was observed [18]. In the present paper, we describe two experimental studies. In an in vivo experiment, we examined the influence of transgenic hIAPP expression on glucose tolerance of mice on a high-fat diet for a long period of time. Previous studies had shown that long-term, high-fat diet induces hyperglycemia, hyperinsulinemia, and obesity in mice [19, 20]. Furthermore, high-fat diet may be involved in islet amyloid formation in hIAPP transgenic mice [21]. We, thus, administered a high-fat diet for 14 months to hIAPP transgenic and nontransgenic (control) mice and report here the islet amyloid formation, glucose tolerance, and islet GLUT-2 mRNA expression. In addition, we describe the development of an ex vivo model system for islet amyloidosis, using pancreatic islets isolated from the hIAPP transgenic mice. When such islets were cultured in high-glucose medium, amyloid formation occurs more rapidly as compared to the in vivo situation. Thus, this ex vivo model system will enable to study the process and effects of islet amyloid formation more specifically and conveniently.

## 2. MATERIALS AND METHODS

### 2.1. Animals

The generation of C57Bl/6J hIAPP transgenic mice with a rat insulin-2 gene promoter fragment (position −695 to +8 relative to the transcription start site) linked to the hIAPP gene has previously been described [15]. The hIAPP transgenic mice were maintained by breeding heterozygous transgenic mice with mates of the C57BL/6J strain. Transgenic mice were differentiated from nontransgenic (NT) littermates by dot blot Southern hybridization, using a 588 bp hIAPP-specific DNA probe [15]. Mice were housed on hardwood bedding in polypropylene cages and maintained in air-conditioned rooms at 20–22°C with a photoperiod of 12 hours light, 12 hours dark. Water was available continuously and the mice received ad libitum a regular diet until 2.5 months of age. This diet contained 4,500 kcal/kg and included 22.5% protein and 4.8% fat (Hope Farms, Woerden, The Netherlands). At 2.5 months of age, the diet was switched to a high-fat diet for 14 months containing 5,600 kcal/kg, 20.8% protein, and 36.0% fat (30.0% cocoa oil, 6.0% corn oil; Hope Farms).

### 2.2. Glucose tolerance test

At 14 months after the start of the high-fat diet, nonfasted mice were anaesthetized with an i.p. injection of midazolam (0.4 mg/mouse) (Dormicum, Hoffman-La-Roche, Basel, Switzerland), and a combination of fluanison (0.9 mg/mouse), and fentanyl (0.02 mg/mouse) (Hypnorm, Janssen, Beerse, Belgium). D-glucose (British Drug Houses, Poole, UK) was injected i.p. (1 g/kg) and blood was sampled from the retrobulbar, intraorbital, capillary plexus before glucose administration and after 10, 30, 60, and 120 minutes. The samples were taken in heparinized tubes and stored on ice. Following centrifugation, plasma was separated and stored at −20°C until analysis. After the 120-minute blood sample, tissue was sampled (see below), and trunk blood was obtained for measurement of IAPP levels. The blood was collected in EDTA-tubes and kept on ice until centrifugation at 1500 g for 5 minutes at 4°C. Plasma was stored at −80°C.

All animal experiments were approved by the Animal Welfare Committee of Utrecht University/University Medical Center Utrecht, The Netherlands.

### 2.3. Plasma measurements

IAPP levels were measured in 25–100 ul plasma by RIA as described [15], using a rabbit, polyclonal hIAPP antiserum (K1338) that shows full cross-reactivity with synthetic amidated rat/mouse IAPP [22]. Free and bound radioactivity was separated by use of double antibody immunoprecipitation. The sensitivity of the assay is 3.5 pmol/l and the coefficiency of variation <10% at both low and high levels. Insulin levels were measured in 20 ul plasma by RIA using guinea pig anti-rat insulin antibody, ^125^I-labelled human insulin as tracer and rat insulin as standard (Linco Research, St. Charles, Mo, USA). Free and bound radioactivity was separated by use of an anti-IgG antibody (Linco). The sensitivity of the assay is 12 pmol/l and the coefficiency of variation <3% at both low and high levels. Glucose was determined in 10 ul plasma by the glucose oxidase method.

### 2.4. Histological analysis of pancreatic tissue

Pancreatic tissue samples were fixed in 3.7% phosphate-buffered formalin (pH 7.4) for 24–48 hours and paraffin embedded. Sections of 5 *μ*m were stained with Congo red for detection of islet amyloid by polarized light microscopy (“apple-green” birefringence) and fluorescence light microscopy (red-coloured autofluorescence). At least 10 islets per mouse were examined. The percentage of individual islet areas occupied by amyloid, as indicated by Congo red positive staining, was visually estimated and scored as follows: 0% = score 0, between 0 and 26% = score 1, 26–50% = score 2, 51–75% = score 3, and 76–100% = score 4. The Amyloid Index (range: 0–100) of an individual mouse was calculated as (1 × *N*1 + 2 × *N*2 + 3 × *N*3 + 4 × *N*4) × 25/*n*, where *N*1 is the number of islets with score 1, *N*2 the number with score 2, and so on, and *n* is the total number of islets investigated. The degree of islet amyloid formation was determined with the investigator being unaware of the genetic status of the animals (i.e., “blind”).

To examine the cellular expression of GLUT-2 mRNA, paraffin sections were subjected to in situ hybridization using a previously described protocol [23] and a ^35^S-labelled oligonucleotide probe covering the nucleotide sequence 247–276 of mouse GLUT-2 cDNA [24]. In order to confirm beta cell expression of hIAPP mRNA in the transgenic mice, sections were also hybridized with a ^35^S-labelled oligonucleotide probe specific for hIAPP mRNA [25].

### 2.5. Image analysis and morphometry

In situ hybridization radiolabelling was examined in a bright field microscope (Olympus, BX60), and images were captured with a digital camera (Olympus, DP50). To quantify the density of labelling for GLUT-2 mRNA within islets, areas of in situ hybridization radiolabelling were calculated. Islets (*n* = 5–8 per animal) were randomly selected from different parts of the sections from 4 mice, 2 males and 2 females in each group. The transgenic mice analyzed were rated as negative for amyloid. The labelled area, that is, grain density within an islet, and total islet area were measured, using NIH-image software, and the density of labelling was expressed as percentage of the total islet area [23, 26]. All sections used were hybridized simultaneously and under identical conditions.

### 2.6. Isolation of pancreatic islets

For islet isolation, transgenic mice were bred to homozygosity for the hIAPP transgenic locus. Homozygotes were discriminated from heterozygous and nontransgenic littermates by dot blot Southern hybridization of tail DNA using a human-specific IAPP probe [15] and quantification of the hybridization signal using phosphor imaging and Image-Quant software (Molecular Dynamics, Inc. Krefeld, Germany).

Islets were isolated from the pancreas of 6-month-old hIAPP transgenic male mice, essentially as previously described [27]. Briefly, under halothane anaesthesia, the abdomen was opened. The pancreas was excised starting from the spleen site to the duodenum. Subsequently, the pancreas was brougth in 10 mL sterile Krebs-Ringer-buffer supplemented with 25 mmol/L Hepes (KRH) and containing 10% Bovine Serum Albumin (BSA) at 4°C. Next, the pancreas was chopped, digested using a two-stage incubation of 20 minutes at 37°C with successively 1.0 and 0.7 mg/mL collagenase (Sigma type XI, Sigma, St Louis, MO, USA). Islets were separated from exocrine tissue by centrifugation over a discontinuous dextran gradient [28] and further purified by handpicking into 9 cm petridishes with 12 mL KRH buffer, pH = 7.4, supplemented with 10% BSA penicillin (100 units/mL)/streptomycin (0.1 mg/mL) (KRH 10% BSA P/S) and glucose to a concentration of 11 mM. Two days after isolation, the islets from 12 mice were pooled, mixed and split into portions. Four portions of 75 islets each were fixed and embedded for amyloid quantification. Eight portions of approximately 90 islets were transferred to culture medium with 28 mM glucose. Medium was changed every 2-3 days, switching between 11 mM and 28 mM of glucose (to prevent possible desensitization of the beta cells). Islets were counted, while being picked into the dishes with fresh medium. At 3 weeks after islet isolation, the cultured islets were fixed and embedded for amyloid quantification.

### 2.7. Fixation and embedding of pancreatic islets

Islets were washed with phosphate buffered saline (PBS), and fixed in 0.5 mL islets fixative (2% paraformaldehyde, 0.2% glutaraldehyde in 0.1 M Sörensen buffer) for 2 hours at room temperature. Fixative was removed and islets were washed with 0.5 mL 0.1 M Sörensen buffer. Sörensen buffer was removed and islets were resuspended in 30 *μ*l 37°C heated 12% gelatin, cooled on ice and stored at −20°C.

### 2.8. Amyloid quantification in cultured islets

From the gelatin-embedded islet blocks, 5 *μ*m frozen sections were cut onto Superfrost Plus microscope slides (Menzel-Gläser) and stored at −20°C until further use. Sections were fixed in acetone for 1′, rehydrated in PBS for 15′, stained with heamatoxylin for 1′, washed in running tap water for 5′, and stained with Congo red (1 g/liter saturated sodium chloride 80% ethanol, into which 10 mL/liter 1% sodium hydroxide was added just before staining) for 30′. After dehydration in an augmenting ethanol series (70%, 96%, 100%) and xylene (twice), sections were enclosed with Depex. Amyloid-containing paraffin sections of hIAPP transgenic mouse pancreatic tissue were used as positive control for the Congo red staining.

For the detection of amyloid, Congo red-stained islet sections were examined using a fluorescence microscope. Amyloid deposits were visible as bright red autofluorescent areas without cells, which showed a green birefringence upon visualization with polarized light. An islet was scored as amyloid positive if at least 2 successive sections of that islet contained Congo red-positive amyloid deposits. The scoring was performed in a “blind” fashion, that is, with the investigator unaware of the source of the islets.

### 2.9. Statistical analysis

Values are means ±SEM, unless stated otherwise. *P*-values indicate the probability level of random difference between groups, or of random correlation, respectively. *P*-values <.05 were considered to represent statistical significance. Nonparametric T-tests were used to compare 2 independent samples (Mann-Whitney-U test: hIAPP versus NT, male versus female). Data of the mRNA in situ hybridizations were analyzed by Student's unpaired t-test. Differences in percentage of amyloid-positive islets between 2 days and 3 weeks of culture were analyzed by use of one-way analysis of variance (ANOVA). Probability values of less than .01 were considered significant.

## 3. RESULTS

### 3.1. Body weight and plasma IAPP levels

Body weight after 14 months on the high-fat diet did not differ between the groups, being 57 ± 0.8 g versus 58 ± 0.9 g in male hIAPP (*n* = 8) and NT (*n* = 5) mice, and 59 ± 1.1 g versus 64 ± 3 g in female hIAPP (*n* = 11) and NT (*n* = 6) mice. Plasma IAPP levels were 462 ± 78 pmol/L in male hIAPP mice versus 195 ± 32 pmol/L in male NT mice, and 346 ± 81 pmol/L in female hIAPP mice versus 130 ± 21 pmol/L in female NT mice, being significantly higher in hIAPP mice of both genders (*P* < .01) without any gender difference.

### 3.2. Glucose tolerance test

**Figure 1:**
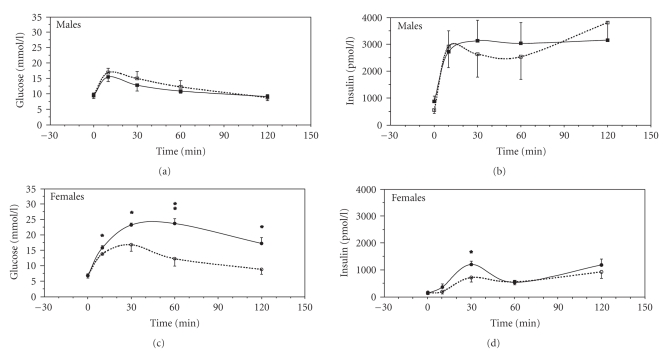
Plasma insulin and glucose levels immediately before and at different timepoints after an intraperitoneal injection of glucose (1 g/kg body weight) in anaesthetized, nonfasted nontransgenic (NT, dotted line), and hIAPP transgenic mice (solid line) on a high-fat diet for 14 months. Mean values and SEM are shown; *n* = 5–11 per group of mice; statistically significant changes between hIAPP and NT mice are indicated by *(*P* < .05) and **(*P* < .01).

After 14 months on the high-fat diet, nonfasted plasma glucose and insulin levels were not different between hIAPP and NT mice of the same gender. However, both for the hIAPP and NT mice, plasma insulin levels were higher in males as compared to females (Figure 1). When glucose was administered i.p. (1 g/kg), the insulin response to glucose and the glucose elimination were similar in hIAPP and NT male mice. In contrast, in female hIAPP mice, plasma glucose levels after the i.p. glucose challenge were markedly higher at all time points as compared to female NT mice (*P* < .05 or *P* < .01) in association with increased insulin levels 30 minutes after glucose administration (*P* < .01). Hence, hIAPP overproduction was associated with severe impairment of glucose elimination in female but not in male mice after high-fat diet.

### 3.3. Pancreatic islet amyloid formation

Islet amyloid was detected in 4/19 hIAPP mice on high-fat diet but in none of the 11 NT mice. The Amyloid Index for these 4 mice was 11.0 ± 6.2 (average and SD). There was no gender difference in islet amyloid formation in hIAPP transgenic mice (3/8 in males versus 1/11 in females).

### 3.4. Islet GLUT-2 mRNA expression

**Figure 2:**
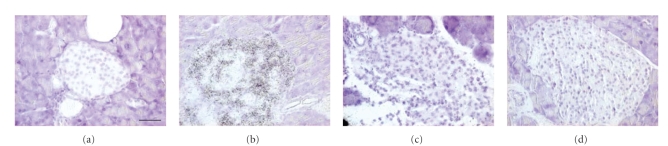
In situ mRNA hybridization (using radiolabeled oligoprobes) for hIAPP (a), (b), and GLUT-2 (c), (d) in islets of nontransgenic (a), (c), and hIAPP transgenic (b), (d) female mice after 14 months on high-fat diet. Note that hIAPP mRNA expression is absent in the nontransgenic islet, and that GLUT-2 mRNA expression is reduced in the transgenic islet. Scale bar = 30 *μ*m.

**Figure 3:**
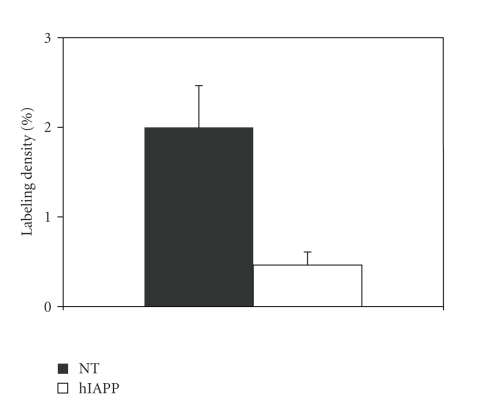
Comparison of the average labeling density of GLUT-2 mRNA in situ hybridization in pancreatic islets from nontransgenic (NT) and hIAPP transgenic (hIAPP) mice (*P* = .02). For both groups 4 mice were analysed, 2 males, and 2 females. The 4 transgenic mice did not have amyloid.

As expected, a strong hIAPP mRNA labeling was observed in the islets of all transgenic mice, while it was lacking in all NT mice (Figures 2(a), 2(b)). GLUT-2 mRNA labeling of weak to moderate density was observed in the islets of NT mice (Figure 2(c)), with no overt difference between female and male mice. In the transgenic mice, however, the GLUT-2 mRNA labeling was generally weaker, and even barely detectable in some female mice (Figure 2(d)). The GLUT-2 mRNA signal was reduced in all transgenic mice, regardless of the presence or absence of islet amyloid. Image analysis revealed a significant reduction of GLUT-2 mRNA labeling of islets in hIAPP transgenic versus NT mice (*P* = .02, Figure 3).

### 3.5. Ex vivo survival and amyloid formation in cultured hIAPP transgenic pancreatic islets

**Figure 4:**
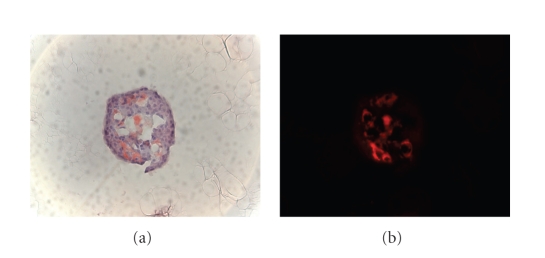
Detection of islet amyloid in islet of Langerhans isolated from an hIAPP transgenic mouse, and cultured in medium with a high glucose concentration. Frozen section of a gelatine-embedded islet was stained with the amyloid-specific dye Congo red and visualized with light microscopy (a) and fluorescence microscopy (b), respectively.

The percentage of 3-week survival of hIAPP transgenic islets was 83.8 ± 1,0% (*n* = 8). Of all islet cultures, 22–30 islets were scored for the presence of amyloid. The percentage of amyloid-positive islets significantly increased (*P* < .001) from 5.5 ± 3.4% (*n* = 4) after 2 days of culture to 70 ± 3.1% (*n* = 8) at the end of the culture period. Thus, the percentage of amyloid-positive islets increased more than 10 times in three weeks of culture at high glucose conditions in this ex vivo islet amyloidosis system. An example of a cultured hIAPP transgenic islet containing amyloid is shown in Figure 4.

## 4. DISCUSSION

### 4.1. High-fat diet and amyloid formation

In this study, transgenic mice overproducing the amyloidogenic hIAPP in their pancreatic islet beta cells, as well as NT control mice, were fed a high-fat diet for 14 months, in order to evaluate the impact on islet amyloid formation and glucose homeostasis when combining these two potentially diabetogenic factors. We anticipated a marked islet amyloid formation in the hIAPP transgenic mice on the high-fat diet because we previously observed that crossbreeding the hIAPP transgenic mice with the Obese mouse (being severly insulin resistant) resulted in extensive islet amyloid formation [18]. Also, when insulin resistance was induced in hIAPP mice by crossbreeding with the obese Agouti viable yellow mice [29] or by exogenous growth hormone and glucocorticoids [30], islet amyloid formation was promoted. In addition, high-fat feeding induced islet amyloid formation in approximately 80% of male mice in another hIAPP transgenic colony [21]. However, we found that only four out of the 19 hIAPP mice (approx. 40% of the males) that were followed for 14 months on the high-fat diet did develop islet amyloid. This lower frequency might be due to differences in the genetic background and/or the composition of the diet, influencing insulin resistance and IAPP expression. The amyloid index in those four high-fat fed mice was higher than in six of 33 similarly aged hIAPP mice (approx. 30% of the males) which developed amyloid on a regular diet (11.0 ± 6.2 versus 4.2 ± 2.9, *P* = .024) [18]. This indicates that although long-term, high-fat diet indeed has the capacity to promote islet amyloid formation in these hIAPP transgenic mice, the efficiency is not high. Crossbreeding the hIAPP mice with leptin-deficient Obese mice introduced more severe obesity and insulin resistance [18] as compared to the high-fat diet. Consequently, these other factors seem of importance for the promotion of islet amyloid formation. Another factor might be hyperglycemia, which is more severe in the hIAPP ob/ob mice as compared to the hIAPP mice on high-fat diet. Such a hypothesis is supported by the finding that in isolated pancreatic islets of our hIAPP mice, islet amyloid was detected by electron microscopy after culture in high-glucose medium but not in low-glucose medium [31]. Other mechanisms may, however, also be of importance.

### 4.2. High fat diet and glucose tolerance

In this study, we also observed severe glucose intolerance in female but not in male hIAPP transgenic mice. The finding that in all groups of mice plasma insulin levels failed to return to basal within 2 hours after the glucose load is in accordance with high-fat diet inducing insulin resistance [32]. Also, the higher insulin levels in male mice versus female mice is well known from previous studies [33]. Thus, our data indicate that the overproduction of insulin in response to insulin resistance after high-fat diet was adequate in hIAPP males but not in hIAPP females. Since there was no gender difference in the amyloid formation in high-fat fed hIAPP mice, these results suggest that a metabolic impact of high levels of circulating IAPP underlies the gender difference in glucose tolerance of hIAPP transgenic mice after high-fat diet. IAPP has, thus, been shown to inhibit insulin secretion [34, 35] as well as to inhibit glycogen synthesis in rat muscle tissue [36] through inhibition of glycogen synthase and stimulation of glycogen phosphorylase [37]. In addition, it has been observed that IAPP administration induces insulin resistance in rats [38], although no such effect was evident in humans [39]. IAPP has also in some studies [40] but not in others [41] been shown to increase liver glucose production. Whether these actions show gender differences, and thus may explain the remarkable glucose intolerance observed in female but not in male hIAPP transgenic mice on the high-fat diet, is not known. Indeed, it is striking that although male rodents generally are more prone to insulin resistance than females, the hIAPP transgenic females on high-fat diet are more glucose intolerant than their male littermates. Since insulin levels are not lower in the hIAPP females compared to the NT females, these data suggest that insulin sensitivity is impaired in the female hIAPP mice.

### 4.3. High-fat diet and GLUT-2 expression

Islet GLUT-2 mRNA expression was reduced in hIAPP transgenic versus NT mice, and this reduction appeared more severe in female than in male mice. Beta cell GLUT-2 expression is known to correlate with glucose responsiveness of the cells [42]. However, insulin levels were not reduced in the male or female hIAPP mice. Therefore, it is presently not known if and how the reduced GLUT-2 mRNA expression among the transgenic mice might be related to the glucose intolerance in the female hIAPP mice. Also, the mechanism of hIAPP mediated downregulation of islet GLUT-2 mRNA expression is unknown, but our data indicate that in addition to inhibition of insulin action in muscle [33, 34] IAPP can (in) directly inhibit glucose responsiveness of islet beta cells by affecting GLUT-2 expression.

In conclusion, this in vivo study shows that promoting insulin resistance over a long period of time by giving a high-fat diet for 14 months promotes islet amyloid formation in hIAPP transgenic mice, although less extensively than in the severe insulin-resistant Obese, leptin-deficient hIAPP mice. This suggests that the degree of insulin resistance is important for extensive development of islet amyloid. In addition, we observed a remarkable gender difference in that severe glucose intolerance was observed only in female hIAPP transgenic mice given high-fat diet and not in males. We suggest that this gender difference is due to the high level of circulating IAPP rather than to islet amyloid formation. If and how this glucose intolerance might be mediated by downregulation of beta cell GLUT-2 gene expression, as observed in the hIAPP mice, is presently unknown.

### 4.4. Ex vivo islet amyloidosis model

The rationale for the ex vivo study was to examine if amyloid would be formed in isolated and cultured pancreatic islets from hIAPP mice, to such a degree that it would be detectable with light microscopy. Since both the present and previous [18] in vivo data indicated the development of islet amyloid notably in male hIAPP mice, we decided to investigate amyloid formation in such islets specifically from male mice. To increase the potential for amyloid formation, we bred the mice to homozygosity for the hIAPP transgene. At an age of 6 months, homozygous transgenic hIAPP males had islet amyloid in about 5% of their pancreatic islets, at 2 days after islet isolation. Previously, we detected amyloid fibrils by electron microscopy in islets from 4–10 months old heterozygous hIAPP transgenic mice, cultured for 1 week in medium with 11 or 28 mM glucose [31]. With the present model, using islets from 6-month-old homozygous hIAPP transgenic males, cultured in medium with high glucose (switching between 11 and 28 mM), we can detect amyloid deposits with Congo red staining and light microscopy, thus enabling quantification of the degree of islet amyloidosis. The number of amyloid-positive islets increased more than 10 fold (from 5.5 to 70%) after 3 weeks of culture in medium containing a high glucose concentration. Although an accurate comparison between the degrees of islet amyloid formation in vivo and ex vivo was not made, our data certainly indicate stronger islet amyloid formation in hIAPP islets cultured ex vivo as compared to in vivo. This might be explained by the higher glucose concentrations in the ex vivo system. hIAPP transgenic mice in vivo have normal plasma glucose concentrations [15, 18], whereas ex vivo the glucose concentration in the medium switched between 11 and 28 mM. It is known that a high glucose concentration triggers both insulin and IAPP secretion, and the hIAPP transgene is under control of an insulin promoter. In addition, macrophages have been implicated in in vivo removal of (beginning) amyloid deposits [43] and such macrophages are absent in the ex vivo system, potentially allowing increased amyloid formation. When combined with a more accurate amyloid quantification procedure involving image analysis, this ex vivo system may present a fast and convenient model to study the process (and factors involved) of islet amyloidosis, as well as the detrimental consequences for individual beta cells (apoptosis) and islet function (insulin producing capacity). In addition, such a model system might be used as an amyloidosis assay to assess the potency of known and novel therapeutic strategies, aimed at reducing, or even preventing, islet amyloid formation, and its effects on beta cell and islet function.
